# A co-culture system to study the effects of Poly I:C-activated microglia on the differentiation of murine primary neural stem cells

**DOI:** 10.1007/s11626-025-01091-6

**Published:** 2025-09-30

**Authors:** Marie Pierre Manitz, Karina Violou, Malin Hedstück, Kimberly Bösing, Maria Kottmann, Nadja Freund, Georg Juckel

**Affiliations:** Division of Experimental and Molecular Psychiatry, Department of Psychiatry, Psychotherapy and Preventive Medicine, LWL University Hospital, Ruhr-University Bochum, Alexandrinenstr. 1, 44791 Bochum, Germany

**Keywords:** Neuronal stem cells, Microglia, Co-culture, Poly I:C

## Abstract

Studies in rodents have shown that systemic inflammation induced by prenatal exposure to the viral mimetic polyinosinic:polycytidylic acid (Poly I:C) triggers maternal immune activation. Cytokines released by the maternal immune system can cross the placenta and enter fetal circulation. In the fetal brain, embryonic microglia may produce additional cytokines and other inflammatory mediators in response to maternally derived cytokines. This resulting cytokine imbalance is suggested to impair neurogenesis and brain development, potentially contributing to the onset of neuropsychiatric disorders in offspring. To investigate microglial involvement in neurogenesis under pathological conditions, we used the spontaneously immortalized microglial cell line (SIM-A9), and confirmed the expression of Iba1 and CD68 via immunocytochemistry. Additionally, SIM-A9 cells expressed CX3CR1, Ki67, and isolectin. Upon Poly I:C stimulation, SIM-A9 cells released the cytokines interleukin-6 (IL-6) and tumor necrosis factor-alpha (TNF-α), as well as nitric oxide (NO), as determined by ELISA and Griess assay, respectively. After confirming SIM-A9 cell activation by Poly I:C, we co-cultured these cells with neural stem/progenitor cells (NSPCs) from embryonic mouse neocortex using a transwell system. We examined how chronically activated microglia influence NSPC differentiation and characterized the resulting cell phenotypes using immunocytochemistry. Our results demonstrate that SIM-A9 cells support NSPC differentiation into neurons as early as three days in culture. However, the number of neurons decreased with prolonged culture. Furthermore, Poly I:C in the NSPC culture media, as well as cytokines secreted by Poly I:C-activated SIM-A9 cells, showed a supportive effect on astrocyte differentiation.

## Introduction

The development of the cerebral cortex involves a series of complex sequential processes including cell proliferation, cell migration, cell differentiation, cortical organization, and the formation of neuronal networks. The cerebral cortex is organized into six distinct layers. The formation of the individual layers relies on neurons that are progressively generated from radial glia cells (RGCs) and intermediate progenitor cells (iPCs), processes referred to as direct and indirect neurogenesis, respectively. RGCs, also known as neural stem cells (NSCs), and iPCs are located in specific niches: the ventricular zone (VZ) and the subventricular zone (SVZ), respectively. In mice, neurogenesis begins at embryonic day (E)9. Around E14, RGCs transition into a glioblast state, giving rise to astrocytes and oligodendrocytes shortly before birth (Kriegstein and Alvarez-Buylla 2009). Environmental insults during early developmental stages can affect neural development (Patterson 2002). Defects in neurogenesis have been implicated in the pathogenesis of neurodevelopmental disorders, such as autism spectrum disorders and schizophrenia (McEwan *et al*. 2023).

NSCs can be isolated from *in vivo* niches of brain tissues (Temple 1989; Moore and Lemischka 2006). They possess the ability to self-renew (Morrison *et al*. 1997) and are multipotent. In vitro, they can form neurospheres (Reynolds and Weiss 1992), which have the capacity to differentiate into all three major cell types of the central nervous system (CNS): neurons, astrocytes, and oligodendrocytes (Gage 2000; Irvin *et al*. 2003; Glaser *et al*. 2007). They are used in both basic and medical research to investigate disease mechanisms and to develop novel therapeutic strategies. Microglia, the immune cells of the brain, play an essential role in CNS development, homeostasis, and pathology. Under physiological conditions, microglia in their resting state actively survey their microenvironment (Davalos *et al*. 2005; Nimmerjahn *et al*. 2005). In response to external cues, they can polarize into two opposite phenotypes: M1 (classically activated) and M2 (alternatively activated). M1 microglia produce and release pro-inflammatory cytokines, chemokines, nitric oxide, reactive oxygen species (ROS), and Prostaglandin E2 (PGE2), and exhibit changes in their morphology, mobility, proliferation rate, antigen presentation, and phagocytic activity (Streit und Kincaid-Colton 1995; Orihuela *et al*. 2016). Conversely, M2 microglia release anti-inflammatory mediators, secrete neurotrophic factors that support cell survival (Garden and Möller 2006) and promote tissue repair (Kreutzberg 1996; Colton 2009; Tang and Le 2016). However, between these two extremes exists a large continuum of microglial phenotypes dependent on the specific stimulus and the CNS microenvironment (Hanisch and Kettenmann 2007; Ransohoff 2016; Paolicelli *et al*. 2022).

Microglia are present throughout all stages of brain development. In the mouse embryo, starting at E8.5, erythromyeloid progenitors emerge in the yolk sac, and colonize the neuroepithelium by E9.5. Microglial progenitors can enter the brain by crossing the blood vessel wall until the blood brain barrier forms at E16.5 (Ginhoux *et al*. 2010; Hattori *et al*. 2022). They populate the brain through migration and proliferation (Smolders *et al*. 2019). They undergo a stepwise maturation process, transitioning from early microglia (until E14) to pre-microglia (E14 to a few weeks after birth) and finally to adult microglia (a few weeks after birth onward) expressing genes in a developmental stage-dependent manner to finally regulate adult brain homeostasis (Matcovitch-Natan *et al*. 2016). Initially, they are ubiquitously distributed. However, from E15 to E16, they are absent from the cortical plate (CP) after migrating to the VZ and SVZ. By E17, they re-enter the CP (Cunningham *et al.* 2013; Swinnen *et al.* 2013; Hattori et al. 2020). They initially exhibit amoeboid or unipolar morphology (Cunningham *et al*. 2007) and gradually acquire a ramified morphology as the CNS matures (Swinnen *et al*. 2013). Recent studies reveal numerous functions of embryonic microglia, including promoting neural progenitor differentiation, regulating progenitor number through phagocytosis, modulating interneuron positioning and wiring, faciliting circuit formation, and regulating gliogenesis and vascularization (Hattori 2022). Environmental perturbations during critical stages of brain development can perturb their physiological functions, leading to impaired neuronal development and an increased risk of neurodevelopmental psychiatric disorders in offspring later in life (Loewen *et al*. 2023; McEwan *et al*. 2023).

SIM-A9 is a microglial cell line that was derived from postnatal murine cerebral cortex tissues. These cells express microglial specific markers such as Iba1 and CD68 (Nagamoto-Combs *et al*. 2014) and respond to exogenous pro- and anti-inflammatory stimuli, including LPS, β-amyloid, ATP, IL-1ß, and IL-4 (Nagato-Combs *et al*. 2014; Farrell *et al*. 2016; Dave *et al*. 2020; Jayakumar *et al*. 2022; Xu *et al*. 2022; de Dios *et al*. 2023). Upon stimulation with LPS, they secrete inflammatory mediators such as TNF-α, IL-1ß, Il-6, NO, MCP-1, MIP2, MIP-1alpha, MIP-1ß, and GS-CSF (Nagamoto-Combs *et al*. 2014; Farrell *et al*. 2016; Jayakumar *et al*. 2022). Stimulation with IL-4 increases arginase (Arg) 1 expression (Nagamoto-Combs *et al*. 2014). This demonstrates that SIM-A9 cells can switch between the pro- (M1) and the anti-inflammatory (M2) phenotypes depending on the nature of the stimulus. Transforming growth factor-beta (TGF-β) promotes SIM-A9 cell proliferation (Bureta *et al*. 2019). Additionally, SIM-A9 cells exhibit phagocytic uptake of fluorescently labeled β-amyloid and bacterial bioparticles (Nagamoto-Combs *et al*. 2014). These findings demonstrate that SIM-A9 cells possess microglial characteristics similar to those of primary microglia, making this cell line optimal to study the effects of inflammation on neural development and the resulting pathologies.

In this study, immunocytochemistry was used to confirm and further characterize the expression of various cell markers in SIM-A9 cells. Poly I:C was then tested as an immunostimulant to activate SIM-A9 cells. The expression and secretion of pro-inflammatory mediators were analyzed using immunocytochemistry, ELISA and Griess assay. Finally, Poly I:C-activated SIM-A9 cells were co-cultured with primary embryonic murine NSPCs in a transwell system to investigate their effect on NSPC differentiation.

## Materials and methods

### Animals

The study was performed in accordance with the Council Directive of the European Parliament and the Council of 22 September 2010 (2010/63EU) on the protection of animals used for scientific purposes and approved by the LANUV (Landesamt für Natur, Umwelt und Verbraucherschutz, North Rhine-Westphalia). Female and male C57/BL6 mice were obtained from Charles River (Sulzfeld, Germany), housed in conventional conditions, and maintained under standard laboratory settings (humidity (60–70%) and temperature (21°C)). They were kept on a 12-h light/dark cycle and provided with ad libitum access to water and food. Mice were mated overnight, and the presence of vaginal plug the following morning corresponded to embryonic day (E) 0.5.

### Cell culture/SIM-A9 cells

The SIM-A9 cell line was purchased from Kerafast (END001, Lot. p8”8/2015″, Boston, MA). SIM-A9 cells were maintained in Dulbecco’s Modified Eagle Medium/F12 (DMEM/F12, Capricorn Scientific, Ebsdorfergrund, Germany) supplemented with L-glutamine (Carl Roth, Karlsruhe, Germany), heat-inactivated fetal bovine serum (FBS, 10%, Life Technologies, Carlsbad, CA) and horse serum (HS, 5%, Capricorn Scientific, Ebsdorfergrund). To prevent microbial contamination, the medium was supplemented with penicillin and streptomycin (Pen Strep, Capricorn Scientific, Ebsdorfergrund). The cells were incubated in a humidified 5% CO2 incubator at 37 ± 0.5˚C. Prior to passaging, the cells were washed with Modified Dulbecco’s Phosphate Buffered Saline (DPBS, Capricorn Scientific, Ebsdorfergrund) and were dissociated using enzyme-free Cell Dissociation Buffer containing 1 mM EGTA, 1 mM EDTA, and 1 mg/ml glucose (Sigma Aldrich, St. Louis, MO) in DPBS. For this study SIM-A9 cells from passages 12 to 17 were used.

### SIM-A9 cells stimulation with Poly I:C

Briefly, SIM-A9 cells were cultured to a monolayer with approximately 90% confluence. The culture medium was discarded, and cells were washed twice with DPBS. They were then cultured in serum-free medium and incubated with Poly I:C at concentrations of 10, 20 or 50 μg/ml for 6 or 24 h. Following incubation, cells were washed with DPBS, fixed with 4% paraformaldehyde (PFA, Carl Roth, Karlsruhe), washed again, and the plates were stored at 4°C for subsequent immunocytochemical staining.

### ELISA

SIM-A9 cells were stimulated with Poly I:C at concentrations of 10, 20 or 50 µg/ml as describe above. After 2, 4, 6, and 24 h of stimulation, supernatants (50 µl) were collected from each well and the levels of pro-inflammatory cytokines IL-6 and TNF-α released into the media were quantified using ELISA according to the manufacturer’s protocol (Thermo Fisher Scientific, Waltham, MA IL-6: 88–7064-88, TNF-α: BMS607-3). ELISA plates were coated with a capture antibody diluted in coating buffer (1:250) and incubated overnight at 4°C. The optical density was measured at 450 nm using a microplate reader.

### Nitric oxide determination

Nitric oxide released from Poly I:C-activated SIM-A9 cells (10–100 µg/ml for 24 h) was converted to nitrite in the culture medium. The nitrite concentration was determined using the Griess reagent protocol, following the manufacturer’s instructions (Promega, Fitchburg, WI). The optical density of assay samples was measured at 548 nm using a microplate reader. Nitrite concentrations were calculated from a standard curve generated with known concentrations of sodium nitrite (0–100 µM).

### Preparation and culture of NSPCs

A total of 41 embryos born to 5 individual dams were used in this study. Each of the 5 experiments was conducted independently on different days. On gestational day (GD) 13, the dams were sacrificed by cervical dislocation, and embryos (E13) were removed from the uterus and placed in ice-cold DPBS. The embryonic cerebral cortices were dissected. Blood vessels and meninges were discarded. Cortical tissue from all embryos in a single litter was pooled and dissociated using trypsin–EDTA solution (0.05% trypsin and 1 mM EDTA, Capricorn Scientific, Ebsdorfergrund). The resulting cells were plated at a concentration of 2 × 10^5^/ml in uncoated dishes with NEP complete medium. The NEP complete medium consisted of Neurobasal™ Plus Medium (Life Technologies, Carlsbad, CA) supplemented with B-27™ (50x, Life Technologies, Carlsbad, CA), N2 (100x, Life Technologies, Carlsbad, CA), human recombinant basic fibroblast growth factor (hb-FGF, 10 ng/ml, Sigma Aldrich, St. Louis, MO), recombinant epidermal growth factor (EGF, 100 ng/ml, Sigma-Aldrich, St. Louis, MO), Pen Strep, and L-Glutamine. Neurosphere formation was monitored daily, and the NEP complete medium was replaced every 2–3 days.

### SIM-A9 cells and NSPCs co-culture

**Figure 1.**
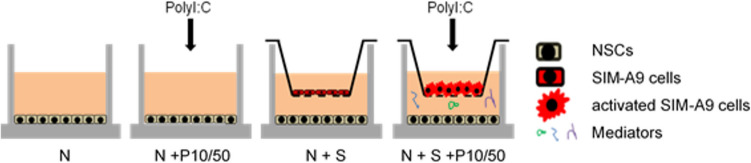
Schematic of experimental design: co-culture of NSPCs and SIM-A9 cells. NSPCs were co-cultured with SIM-A9 cells either for 3 d or 7 d in a transwell-system. SIM-A9 cells were activated with 10 μg/ml or 50 μg/ml Poly I:C to induce production and secretion of inflammatory molecules. NSPCs incubated with Poly I:C (10 μg/ml or 50 μg/ml) and without Poly I:C were used as controls.

To analyze the effects of microglia on NSPCs, co-cultures of SIM-A9 cells and NSPCs were established using Transwell permeable supports as previously described (Farrell *et al*. 2016) and illustrated in Fig. 1. After 6 days in vitro (DIV), primary neurospheres were collected by centrifugation (1100 rpm), gently dissociated into single cells using trypsin–EDTA (0.05%), and plated at a density of 3 × 10^4^ in 200 µl/well in a 24-well plate (Sarstedt,Nümbrecht, Germany) on glass coverslips pre-coated with Poly-L-ornithine (PLO, 50 µg/ml, Sigma Aldrich, St. Louis, MO) and laminin (5 µg/ml, Sigma Aldrich, St. Louis, MO) in differentiation medium (NEP complete medium without hbFGF and EGF). After attachment (approximately 3 h at 37°C), Transwell inserts (TC-Insert, PET 0.4 µm, TL, Sarstedt,Nümbrecht, Germany) were placed inside the wells, and 1.75 × 10^5^ SIM-A9 cells were seeded along with serum-free SIM-A9 cell culture medium containing Poly I:C (Sigma Aldrich, St. Louis, MO) at concentrations of 10 µg/ml or 50 µg/ml. Media for both cell types (NSPCs and SIM-A9 cells) were replaced daily. The experimental setup included 3 different NSPC cultures and 3 different co-cultures (N: NSPCs cultured standalone in differentiation medium; N + P10: NSPCs in differentiation medium containing 10 µg/ml Poly I:C; N + P50: NSPCs in differentiation medium containing 50 µg/ml Poly I:C; N + S: co-culture of NSPCs with SIM-A9 cells; N + S + P10: co-culture of NSPCs with SIM-A9 cells stimulated with 10 µg/ml Poly I:C; and N + S + P50: co-culture of NSPCs with SIM-A9 cells stimulated with 50 µg/ml Poly I:C). NSPCs standalone and NSPCs cultured with Poly I:C served as controls. After either 3 or 7 days of culture, the inserts were removed, and cells were processed for immunostaining.

### Immunocytochemistry

Cells (SIM-A9 cells, Poly I:C-activated SIM-A9 cells, and differentiated NSPCs) were washed with Phosphate Buffered Saline (PBS, VWR, Darmstadt, Germany), fixed with 4% PFA for 10 min, and then blocked for one hour at room temperature in PBS containing 1% bovine serum albumin fraction V (BSA, Sigma Aldrich, St. Louis, MO), 2% fetal bovine serum (FBS, Life Technologies, Carlsbad, CA), and 0.1% Triton X-100 (Electran, VWR Life Science, Darmstadt). SIM-A9 cells were then incubated in the blocking buffer at 4°C overnight with primary antibodies against the following proteins: Iba1 (1:500, FUJIFILM Wako Chemicals, Neuss, Germany, 019–19,741), CD68 (1:500, Bio-Rad, Hercules, CA, MCA1957T), CX3CR1 (Thermo Fisher Scientific, Waltham, MA, 1:700, 14–6093), Ki-67 (1:100, Santa Cruz Biotechnology, Dallas, TX, sc-7846), NeuN ((D4G40),1:100, Cell Signaling Technology, Cambridge, UK, 24307 T), and GFAP (1:500, Merck Millipore, Billerica, MA, MAB360). Poly I:C-activated SIM-A9 cells were incubated overnight with anti-iNOS (1:50, Enzo life Sciences, Farmingdale, NY, ADI-KAS-NO001), anti-Il-6 (1:100, Proteintech, Planegg-Martinsried, Germany, 666,146–1-Ig), and anti CX3CR1 antibodies. Differentiated NSPCs were co-stained with rabbit anti ßIII-tubulin antibody (1:200, Sigma-Aldrich, St. Louis, MO, T2200) for neurons and GFAP for astrocytes at 4°C overnight. After incubation, cells were rinsed three times with PBS and then incubated with appropriate secondary antibodies: CF™ 555-conjugated goat anti-rabbit IgG (1:1000, Sigma-Aldrich, St. Louis, MO, GSAB4600068), CF™ 555-conjugated goat anti mouse IgG (1:1000, Merck, Darmstadt, Germany, SAB4600066), FITC-conjugated goat anti-mouse IgG (1:500, Sigma-Aldrich, St. Louis, MO, AQ303F), Alexa Fluor 488-conjugated Goat anti-Rat IgG (1:500, abcam, Cambridge, UK, ab150157), and CF 488A-conjugated goat anti-rabbit IgG (1:1000, Merck, Darmstadt, Germany, SAB4600044) at room temperature for 1.5 h. For direct staining, fluorescein phalloidin (1:40, Invitrogen, Carlsbad, CA, F432) and FITC-conjugated isolectin (1:100, Sigma-Aldrich, St. Louis, MO, L2895) were used. Cells were then rinsed with PBS and stained with 0.5 µg/ml DAPI (Sigma-Aldrich, St. Louis , MO, D8417) before being mounted with Mowiol 4–88 (Carl Roth, Karlsruhe, Germany, 0713.2).

### Cell counting and statistics

The number of positive cells expressing a given marker was determined relative to the total number of DAPI-labeled nuclei. At least 300 cells per well were counted for the immunostaining of SIM-A9 cells, and at least 500 cells per condition were counted for the immunostaining of NSPCs after differentiation in each experiment. Statistical analyses were performed using IBM SPSS Statistics software (version 27). Data were tested for normal distribution using the Shapiro–Wilk-test, which revealed that the data were not normally distributed. The Chi-quadrat test and Bonferroni post-hoc test (to determine which means were significantly different) were used for statistical analysis. Data are presented as mean ± SEM, and *p* values < 0.05 considered statistically significant.

## Results

### SIM-A9 cells expressed microglia-specific proteins and displayed diverse morphologies

**Figure 2.**
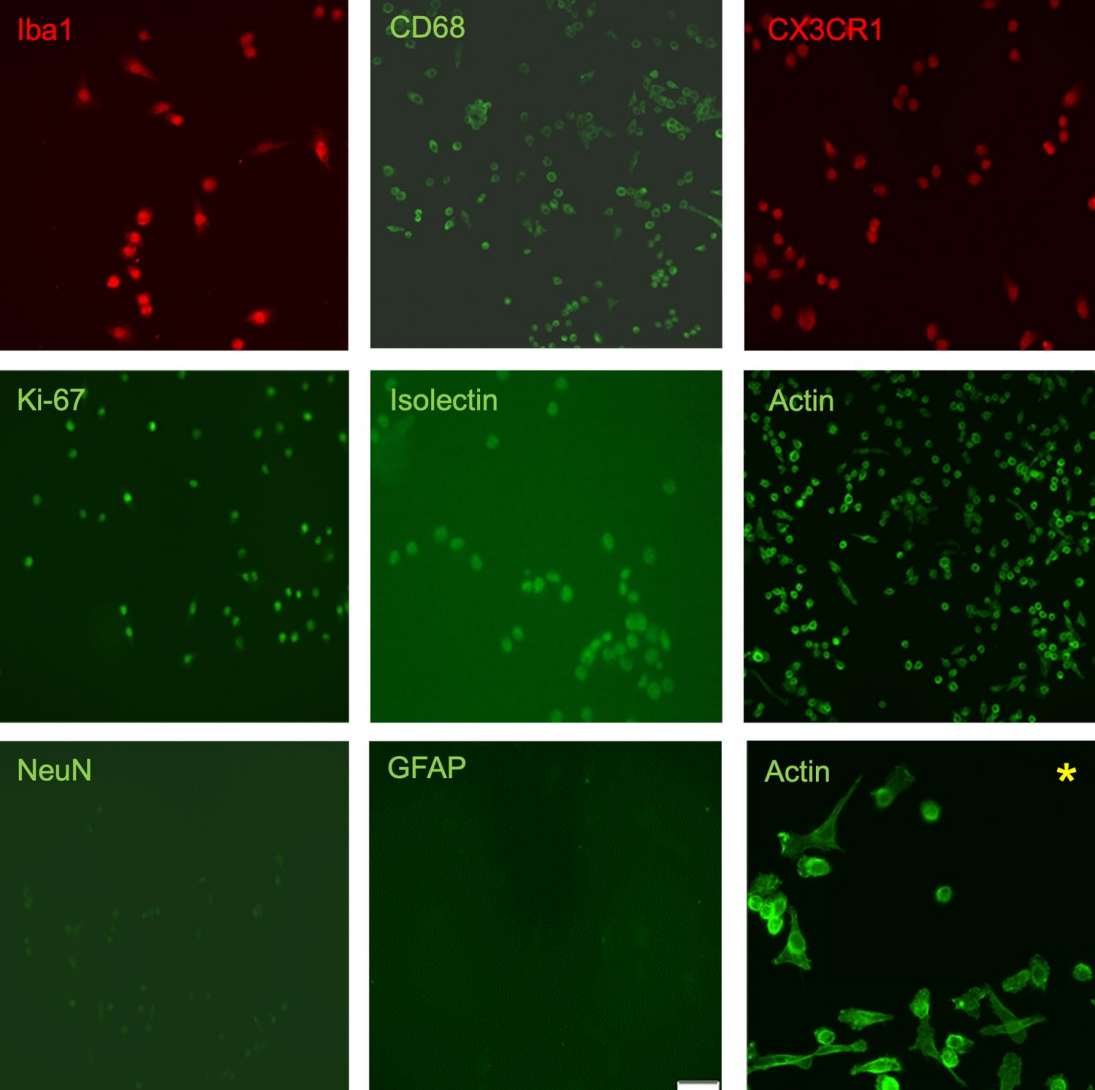
SIM-A9 cells expressing microglia-specific (Iba1, CD68, and CX3CR1) and non-specific (Isolectin, Actin, and Ki-67) proteins. Immunostaining was performed with SIM-A9 cells (passages 12–14) after 24 h culture. Magnification 200x. There was neither an expression of NeuN nor GFAP. Staining with phalloidin shows the different morphologies of SIM-A9 cells in culture. Asterisk: magnification 400x. Representative fluorescence images of 3 independent experiments.

SIM-A9 cells were examined for the expression of various microglial marker molecules. Immunocytochemical (ICC) analysis confirmed the expression of Iba1 and CD68, and the absence of GFAP and NeuN, as previously reported by Nagamoto-Combs *et al*. (2014). Additionally, ICC staining revealed that SIM-A9 cells cultured for 24 h in growth medium expressed the fractalkine receptor CX3CR1 and isolectin. Staining with an anti Ki-67 antibody indicated the proliferative activity of SIM-A9 cells. Phalloidin staining demonstrated that SIM-A9 cells exhibited diverse morphologies in culture. Most cells displayed an amoeboid-like (rounded) shape without distinct processes, while others were bipolar or polygonal as shown in Fig. 2.

### Effects of Poly I:C on cellular expression of IL-6, iNOS, and morphology in SIM-A9 cells

To investigate the stimulatory potential of Poly I:C in SIM-A9 cells, the expression kinetics of inducible nitric oxide synthase (iNOS) and IL-6 were analyzed. Additionally, cellular morphology was observed using phalloidin and CX3CR1 staining.

**Figure 3.**
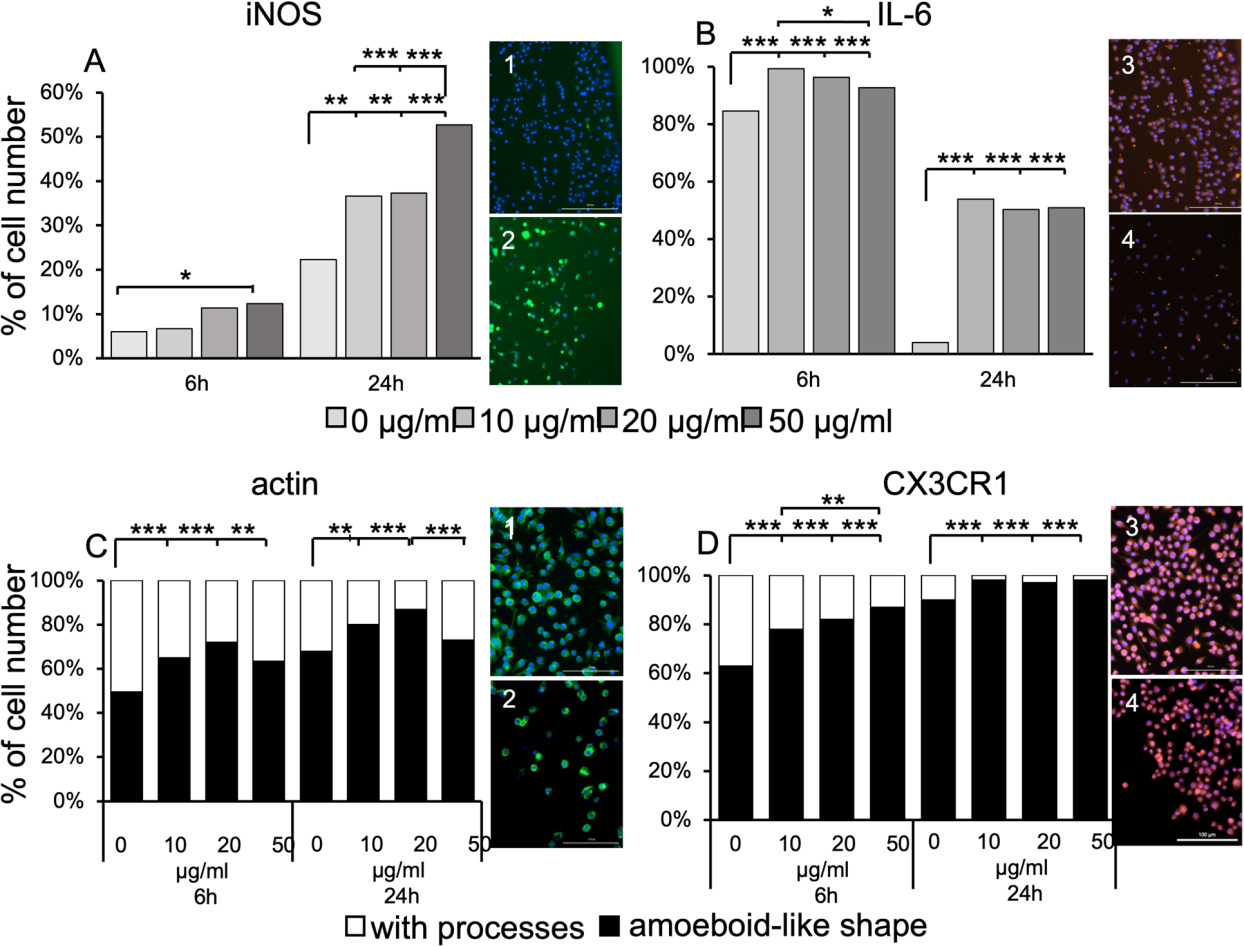
Differential expression and morphological changes in SIM-A9 cells in response to Poly I:C. Cells were exposed to different concentrations (10, 20, and 50 µg/ml) of Poly I:C for 6 and 24 h (h). Nuclei were stained with DAPI (*blue*). *A*: Percentage of SIM-A9 cells stained positive for iNOS (*green*). An increase in iNOS expression was observed in a concentration- and time-dependent manner. a1: SIM-A9 cells exposed to 10 µg/ml Poly I:C for 6 h, showing few or weakly iNOS-positive cells. a2: SIM-A9 cells exposed to 50 µg/ml Poly I:C for 24 h, showing many iNOS-positive cells. *Scale bar* = 200 µm *B*: Percentage of SIM-A9 cells positive for IL-6 (red). Prolonged culture led to a decrease of IL-6 expression in SIM-A9 cells. After 6 h, Poly I:C caused an increase in IL-6 expression with the highest IL-6 expression observed at 10 µg/ml Poly I:C). b3: SIM-A9 cells exposed to 10 µg/ml Poly I:C for 6 h, showing IL-6-positive staining. b4: SIM-A9 cells exposed to 20 µg/ml Poly I:C for 24 h, showing few or weakly IL-6-positive cells. *Scale bar* = 200 µm. *C*: Percentage of SIM-A9 cells stained for actin with phalloidin (green), showing amoeboid-like morphology (rounded cells without obvious processes; *black*) or microglial processes (*white*). Upon Poly I:C stimulation, the number of amoeboid-like SIM-A9 cells increased in a time- and dose-dependent manner, except at the highest concentration. After 24 h of Poly I:C stimulation, the highest number of rounded SIM-A9 cells was observed at 20 µg/ml Poly I:C. Cells exposed to 50 µg/ml Poly I:C showed a higher proportion of rounded cells, but after 24 h, the difference was no longer significant compared to unstimulated cells. c1: Unstimulated SIM-A9 displaying long and short processes cells after 6 h of culture, stained positive for phalloidin. c2: SIM-A9 cells exposed to 20 µg/ml Poly I:C for 24 h, with many amoeboid-like cells stained positive for phalloidin. *Scale bar* = 100 mm. *D*: Percentage of SIM-A9 cells stained for CX3CR1 (red), with an amoeboid-like morphology (rounded cells without obvious processes; *black*) or with microglial processes (*white*). An increase in rounded cells was observed in a time- and dose-dependent manner. d3: SIM-A9 cells exposed to 10 µg/ml Poly I:C for 6 h, showing some cells with processes. d4: SIM-A9 cells exposed to 50 µg/ml Poly I:C for 24 h, showing many rounded cells. *Scale bar* = 100 µm. Experiments were performed in four replicates, with approximately 1200 cells counted per staining. * *p* <.05; ** *p* <.01; *** *p* <.001.

The number of iNOS-positive SIM-A9 cells increased in both a concentration- and time- dependent manner. After 6 h in culture, 6% of untreated SIM-A9 cells expressed iNOS, compared to 53% of cells stimulated with 50 µg/ml Poly I:C at 24 h, as shown in Fig. 3*A*. A chi-square test was performed and indicated a significant relationship between Poly I:C stimulation and iNOS expression at 6 h (χ^2^(3) = 11.251, *p* = 0.01, φ = 0.1) and at 24 h (χ^2^(3) = 59.107, *p* < 0.0001, φ = 0.222).

Furthermore, the application of 10 µg/ml of Poly I:C for 6 h resulted in the highest proportion of IL-6-positive SIM-A9 cells (99%). However, higher concentrations and/or prolonged incubation caused a marked decrease in the number of IL-6-positive cells. At 24 h, only 51% of the cells incubated with 50 µg/ml of Poly I:C still expressed IL-6. A significant association was found between Poly I:C stimulation and IL-6 expression at 6 h (χ^2^(3) = 57.447, *p* = 0.0001, φ = 0.219) and at 24 h (χ^2^(3) = 215.260, *p* < 0.0001, φ = 0.434) (Fig. 3*B*).

Additionally, a Chi-square test was performed to investigate whether there was a statistically significant association between Poly I:C concentration and the rounded morphology of SIM-A9 cells. Results from phalloidin staining revealed a significant relationship between these variables at 6 h (χ^2^(3) = 38.129, *p* = 0.0001, φ = 0.173) and at 24 h (χ^2^(3) = 35.862, *p* = 0.0001, φ = 0.171). Under the condition of 20 µg/ml Poly I:C at 24 h, the highest percentage of rounded cells was observed (Fig. 3*C*). Results from CX3CR1 staining also showed a significant relationship between these variables at 6 h (χ ^2^(3) = 113.015, *p* = 0.0001, φ = 0.192) and at 24 h (χ^2^(3) = 81.183, *p* = 0.0001, φ = 0.148) (Fig. 3*D*). An increase in the percentage of rounded cells was observed in a time- and dose-dependent manner. Post-hoc-Bonferroni-tests were conducted to identify statistically significant results.

### Effects of Poly I:C on the secretion of IL-6 and TNF-α

**Figure 4.**
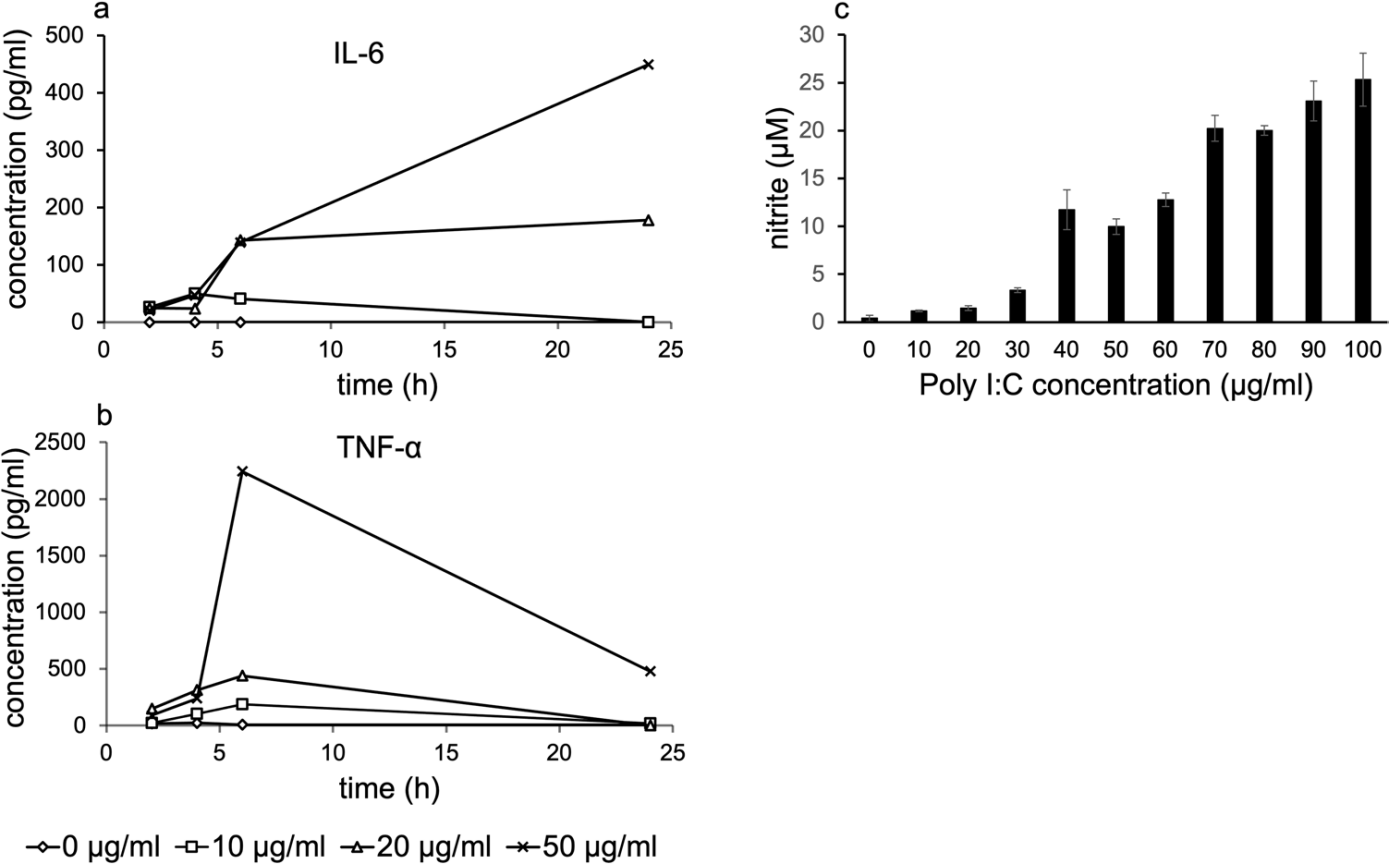
Effect of Poly I:C stimulation on the secretion of IL-6, TNFα, and NO by SIM-A9 cells analyzed using ELISA and Griess Assay. SIM-A9 cells were cultured in 24 well plate and grown to confluency (~ 90%). After washing cell culture medium without serum containing 10, 20, or 50 µg/ml Poly I:C was added to the cells. After 2, 4, 6, and 24 h 50 µl of the supernatant was removed from each well for ELISA analysis. 4a: In the absence of Poly I:C stimulation (0 µg/ml) IL-6 was not detected in the supernatant at any time point. Increasing Poly I:C concentration (20 and 50 µg/ml) led to increased IL-6 concentrations in a time-dependent manner. 4b: Increasing Poly I:C concentration (10, 20 and 50 µg/ml) led to increased TNF-α production and secretion into supernatant. Maximal TNFα-concentrations were measured after 6 h incubation independently of the Poly I:C concentrations. Prolonged incubation times caused a decrease of TNF-α concentrations. 4c: An increased accumulation of nitrite in cell supernatant was observed in a dose-dependent manner. The concentration of NO production was quantified by being plotted against a standard curve. The experiment was replicated three times. Data represent the mean ± SEM

ELISA results demonstrated that IL-6 secretion occurred in a dose- and time- dependent manner, with the highest concentration detected in the supernatant at 24 h following incubation with 50 µg/ml Poly I:C (Fig. 4a). TNF-α secretion was dose-dependent, with levels gradually increasing and peaking at 6 h, followed by a decline thereafter (Fig. 4*b*).

### Effects of Poly I:C on nitric oxid production

The Griess method was used to quantify nitrite levels in culture supernatants. Nitrite was detected at a Poly I:C concentration as low as 10 µg/ml. An increase in NO production was observed in a dose-dependent manner (Fig. 4*c*).

### Differential effects of Poly I:C and SIM-A9 cells on NSPCs differentiation

**Figure 5.**
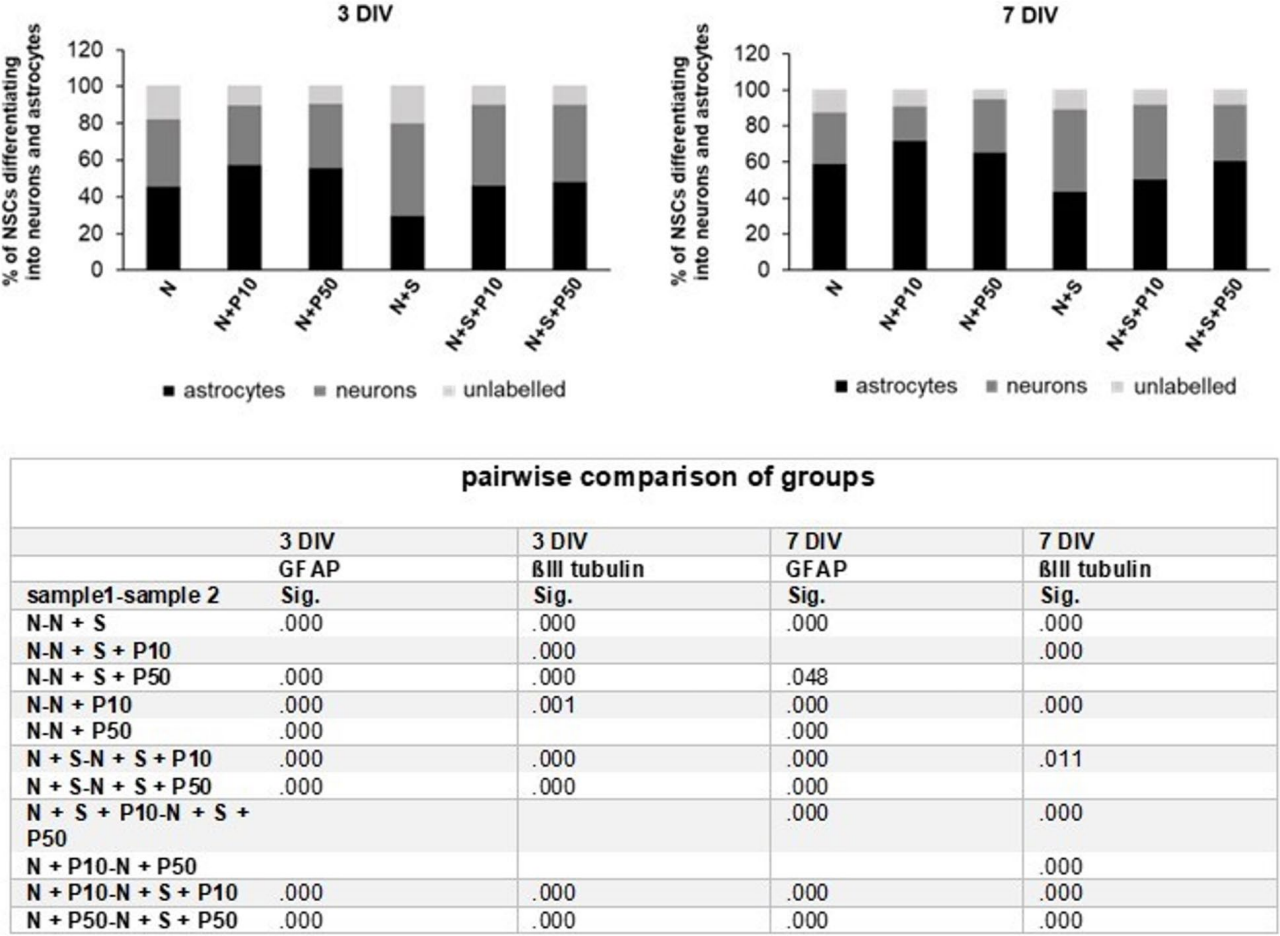
Percentages of neurons, astrocytes, and unlabeled cells after 3 and 7 d of culture. Results were obtained from five independent experiments (5 dams; 41 embryos in total). In each experiment NSPCs were plated in 24-well plates in duplicate for all conditions. Cells cultured for 3 and 7 DIV were stained with anti-GFAP and anti-ßIII-tubulin antibodies to mark astrocytes and neurons, respectively. Cell nuclei were stained with DAPI. A minimum of 500 cells (neurons, astrocytes and unlabeled cells combined) were counted per well. SIM-A9 cells promoted the differentiation of NSPCs into neurons. Poly I:C, at both concentrations and with prolonged culture, promoted the differentiation of NSPCs into astrocytes. Bonferroni post-hoc test was performed to detect differences between the groups of interest. No value = *p* >.05 (see table).

Neurospheres after 6–7 DIV were collected, enzymatically dissociated, and plated as single cells on laminin- and Poly-L-ornithine-coated coverslips. The cells were cultured in differentiation medium (devoid of growth factors) for either 3 or 7 days. Upon the removal of GFs, NSPCs efficiently generate both neurons and glial cells (Glaser *et al*. 2007). To investigate the effect of microglia on NSPC differentiation, three different co-culture conditions were applied: N + S, N + S + P10, and N + S + P50. Standalone NSPC cultures (N, N + P10, and N + P50) served as controls (Fig. 1). As early as 3 days of culture, immunocytochemistry revealed that NSPCs differentiated into neurons and astrocytes. However, the number of neurons decreased with time regardless of conditions (Fig. 5).

A Chi-square test was calculated to investigate whether there was a statistically significant association between culture conditions and NSPC fate decisions. The results of the cross-tabulation indicate a relationship between the two variables Poly I:C and the number of GFAP-positive cells after 3 days of culture. In the N + P10 condition, approximately 58% of the total cells became astrocytes (while 32.5% were neurons). A similar trend was observed in the N + P50 condition, where ~ 56% of the total cells were GFAP-positive astrocytes, and 34.5% were ßIII-tubulin-positive neurons. After 7 DIV, under N + P10 condition, 72% of cells expressed GFAP, while 19,5% expressed ßIII-tubulin. Similarly, under the N + P50 condition, ~ 66% of cells expressed GFAP, while ~ 29% expressed ßIII-tubulin. These results suggest that Poly I:C stimulation promotes the differentiation of NSPCs into astrocytes.

**Figure 6.**
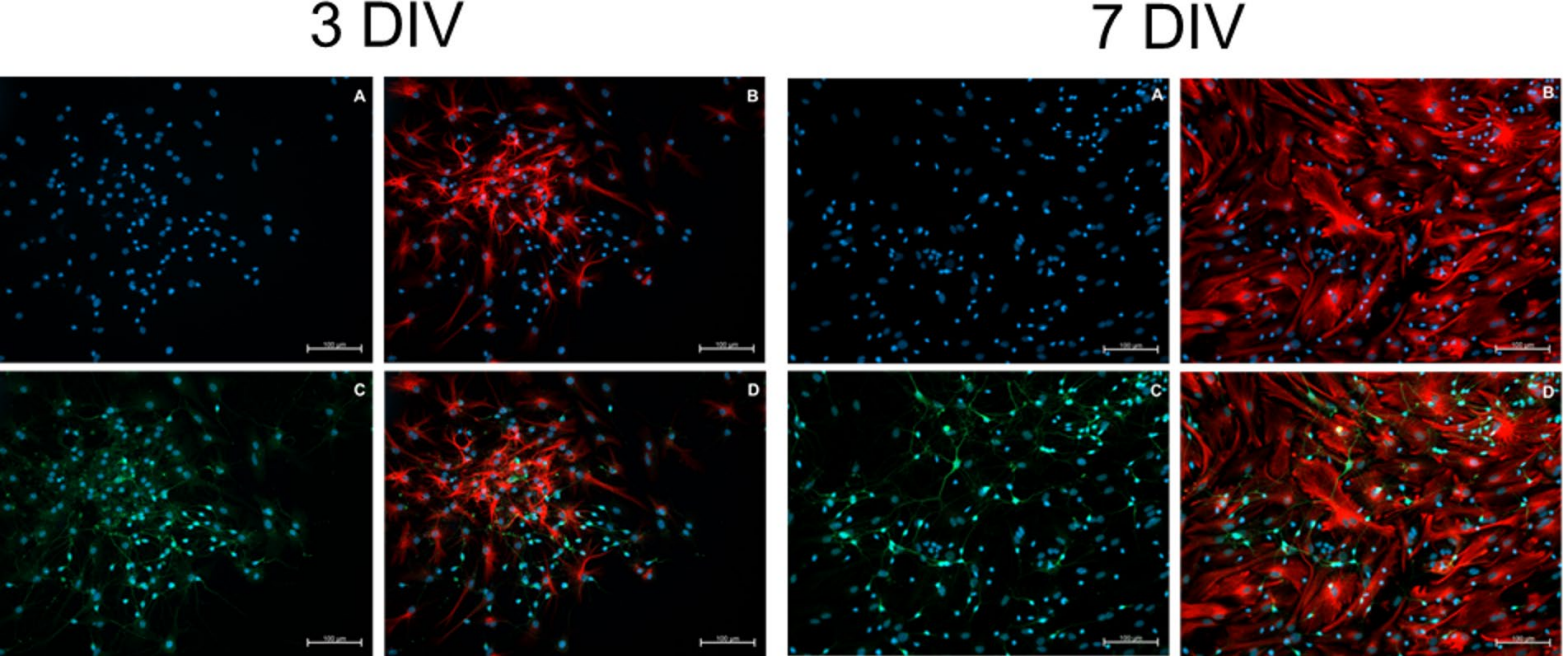
Representative pictures of differentiated astrocytes and neurons derived from NSCs isolated at E13 after three and seven days of co-culture with SIM-A9 cells (N + S). (*A*) Cell nuclei (in blue) stained with DAPI. (*B*) Astrocytes (in *red*) after immunostaining analysis using the anti-GFAP-antibody. (*C*) Neurons (in *green*) after immunostaining analysis using the anti-ßIII-tubulin-antibody. (*D*) merged images (combined *red*, *green*, and *blue*). The highest number of neurons and the lowest number of astrocytes on 3 DIV were observed under condition „N + S “. After 3 DIV, astrocytes displayed an asymmetric morphology with cell processes more or less elongated. After 7 DIV, irrespective of whether they were bipolar or multipolar, they were flattened and extended their surface. This indicates that astrocytes are able to exhibit various cellular shapes in vitro. It seems that despite the presence of SIM-A9 cells, the number of neurons decreased. *Scale bar* = 100 μm.

The results also reveal a relationship between SIM-A9 cells and the number of ßIII-tubulin-positive cells. Under the N + S condition, ~ 50% of cells expessed ßIII-tubulin, while only ~ 30% of cells were GFAP-positive after 3 DIV. Similar results were observed after 7 DIV. Approximately 46% of cells differentiated into neurons, while ~ 44% differentiated into astrocytes (Fig. 5). For example, the differentiation kinetics of astrocytes and neurons under the N + S condition are shown in Fig. 6. After 3 DIV, astrocytes had star-shaped cell-bodies with short and long processes (Fig. 6*B*), whereas neurons had smaller cell bodies with thin axons and short processes (Fig. 6*C*). After 7 DIV, the cell bodies of astrocytes became larger and flatter compared to those observed after 3 d, and their processes expanded further, forming long and wide branches that increased their territory (Fig. 6B). Neurons developed larger cell bodies, and their axons gradually elongated (Fig. 6*C*).

Results of the Chi-square test show a significant association between Poly I:C treatment and NSPC differentiation into astrocytes after 3 DIV (χ^2^(5) = 507.967, *p* < 0.001, φ = 0.177) and 7 DIV (χ^2^(5) = 370.186, *p* < 0.001, φ = 0.192). Additionally, a significant association was observed between the co-culture of SIM-A9 cells with NSPCs and their differentiation into neurons after 3 DIV (χ^2^(5) = 243.895, *p* < 0.001, φ = 0.122) and 7DIV (χ^2^(5) = 343.432, *p* < 0.001, φ = 0.185). Post-hoc-tests were conducted to assess whether the culture conditions differed significantly from each other in mean value. The results are shown in Fig. 5.

## Discussion

The aim of this study was to investigate the effects of inflammatory mediators secreted by Poly I:C-activated SIM-A9 cells on NSPC differentiation. Due to the complexity of the brain, studying these mechanisms in vivo is challenging. Therefore, we performed a series of cell culture experiments using the transwell culture system (Patchos *et al*. 2015). In this system, microglia and NSPCs are physically separated but can interact via secreted factors in the culture medium. This setup allows for the examination of the potential role of microglia in neurotoxicity.

The SIM-A9 cell line expressed microglia-specific markers, such as Iba1 and CD68, while lacking neuronal and astrocytic markers NeuN and GFAP. These findings are consistent with those reported in a previous study (Nagamoto-Combs *et al*. 2014) and were futher validated in our study. Additionally, we demonstrated that SIM-A9 cells express the microglial marker CX3CR1, the proliferation marker Ki-67, and the activation marker isolectin IB4.

Recently, SIM-A9 cells has been used in various inflammatory in vitro studies and stimulated with different agents, such as ß-amyloid and LPS, to analyze their capacity to secrete inflammatory mediators (Farrell *et al*. 2016, Nagamoto-Combs *et al*. 2014), investigate signaling pathways (Dave *et al*. 2020; Kurt *et al*. 2020; Jayakumar *et al*. 2022), and test various substances capable of mitigating microglia-mediated inflammation and their therapeutic potential (Gill *et al*. 2018; Che *et al*. 2020; Tyrtyshnaia *et al*. 2021; Zhang *et al*. 2023). Taken together, SIM-A9 cells exhibit characteristics similar to those of primary microglia, supporting their suitability for investigating microglial interactions with NSPCs. These findings further validate their use as a model for neuroinflammation research. Primary microglia are technically time consuming to prepare and mouse embryos or pups are needed for the procedure. Considering the replacement of animals in research, SIM-A9 cells provide an ideal easy-to-use alternative to primary microglia.

In this study, we investigated the response of SIM-A9 cells to Poly I:C stimulation. Poly I:C binds to the toll-like receptor (TLR) 3, which is expressed by sentinel cells of the innate immune system. This interaction activates various intracellular signaling pathways, leading to antiviral and inflammatory responses. The acute inflammatory reaction is primarily characterized by the secretion of pro- and anti-inflammatory cytokines (Cunningham *et al*. 2007). Previous data suggest that Poly I:C acts as a specific ligand for TLR3 in primary or immortalized microglial cells, such as BV2, at concentrations ranging from 0.1 to 50 µg/ml (Lee *et al*. 2007; de Oliveira *et al*. 2016; Jeong *et al*. 2017; Kichev *et al*. 2017; He *et al*. 2021; Wegrzyn *et al*. 2021). Based on this, SIM-A9 cells were stimulated with 10, 20, and 50 µg/ml of Poly I:C for 6 or 24 h to investigate their potential to polarize towards the M1 phenotype. We first analyzed the morphology of SIM-A9 cells by staining for actin and CX3CR1. Our results showed that an increased Poly I:C concentration up to 20 µg/ml or prolonged culture duration led to a higher number of cells exhibiting an amoeboid like-shape (rounded cells) (Fig. 3*C* and *D*). However, stimulation with 50 µg/ml Poly I:C resulted in a decrease in the number of actin-positive rounded cells, suggesting that this high concentration may inhibit cytoskeletal rearrangement.

Phalloidin staining of untreated SIM-A9 cells revealed a predominantly rounded morphology, with the number of amoeboid-like cells increasing over time (50% after 6 h and 63% after 24 h) as shown in Fig. 3*C*. Similar trends were observed in CX3CR1-stained untreated SIM-A9 cells, where the proportion of rounded cells increased from 63% after 6 h to 90% after 24 h (Fig. 3*D*). The rounded morphology of untreated SIM-A9 cells may indicate an „activation state “. However, morphological variations are currently observed in culture depending on factors such as culture conditions (e.g., serum free medium, uncoated plates) and gene expression patterns (Nagamoto-Combs *et al*. 2014, Bohlen *et al*. 2017). Therefore, changes in microglial morphology alone should not be universally interpreted as a sign of activation (Beyer *et al*. 2000; Harry and Kraft 2012). Notably, the addition of Poly I:C to the culture medium resulted in a significant increase in the number of cells with an amoeboid-like morphology, indicating a robust response to this specific challenge. Furthermore, SIM-A9 cells demonstrated responsiveness to exogenous inflammatory stimulation with Poly I:C, as evidenced by increased expression levels of iNOS and IL-6 (Fig. 3*A* and *B*, respectively). The cytokines IL-6 and TNF-α and nitrite were detected in the supernatants, confirming that Poly I:C-stimulated SIM-A9 cells secreted these mediators (Fig. 4). It is well-established that pro-inflammatory cytokines and NO are hallmark products of microglial activation and play critical roles in mediating inflammatory responses and contributing to cytotoxic damage to surrounding neurons and neighboring cells (Cunningham *et al*. 2007, Field *et al*. 2010). Interestingly, despite the increased secretion of IL-6 into the culture medium, we observed a decrease in the percentage of IL-6-positive SIM-A9 cells as detected by immunocytochemistry (Fig. 3). This apparent discrepancy suggests differential regulation of intra- versus extracellular cytokine dynamics, particularly at higher doses of Poly I:C. One possible explanation is that high-dose Poly I:C stimulation enhances IL-6 secretion, thereby reducing intracellular immunoreactivity while increasing extracellular levels. Alternatively, strong or prolonged inflammatory stimulation may trigger negative feedback mechanisms, such as the induction of suppressor of cytokine signalling (SOCS) proteins, which inhibit cytokine signalling pathways (Yoshimura *et al*. 2007). Activation of the JAK/STAT pathway by cytokine-receptor interaction promotes transcription of SOCS genes, leading to feedback inhibition (Sobah *et al*. 2021). In particular, SOCS3 is known to regulate IL-6 family cytokines signaling through STAT3, thereby limiting excessive IL-6-mediated inflammation (Babon *et al*. 2014). It has been proposed that SOCS3 suppresses M1 microglial activation via inhibition of the JAK2/STAT3 pathway (Baker *et al*. 2009).

These considerations highlight the need to assess both intracellular and secreted cytokine levels to fully understand inflammatory responses.

After confirming that Poly I:C challenge induced an inflammatory response in SIM-A9 cells, we started different co-culture settings to examine the impact of activated microglia on NSPC differentiation. Our results indicate that most cells expressed either TUJ1 or GFAP, regardless of culture conditions, as early as 3 DIV. At this time, 9–19% of the cells were not labeled with these markers. However, the percentage of unlabeled cells decreased over the 7-day period, ranging between 5–12% at 7 DIV, depending on the culture conditions. This reduction likely reflects the continuously division and differentiation of NSPCs over time, while the unlabeled cells either failed to differentiate or did so more slowly. Alternatively, the unlabeled cells may represent earlier stages of neurogenesis and express markers such as nestin and doublecortin (DCX), rather than TUJ1, which is expressed in early postmitotic and differentiated neurons (von Bohlen Und Halbach 2007). Another possibility is that the unlabeled population includes oligodendrocyte progenitor cells (OPCs) or more mature oligodendrocytes. This could be addressed in future studies using immunostaining for markers such as Oligo2, which is expressed across the oligodendrocyte lineage, from OPCs to mature oligodendrocytes (Chew *et al*. 2013).

At 3 DIV, nearly half of the standalone NSPCs had differentiated into astrocytes (~ 46%), while the proportion of neurons was slightly lower (~ 37%). By 7 DIV, ~ 60% of the cells were astrocytes, with neurons constituting only about 30%. This observation contrasts with findings by Farrell *et al*., who reported that NSCs isolated from mouse E11.5 predominantly differentiated into neurons by 10 DIV (Farrell *et al*. 2016). This discrepancy could be attributed to differences in experimental conditions, including developmental stage at isolation (E11.5 vs. E13.5), NSC isolation procedures, culture conditions (e.g. adhesion substrates such as Poly-L-lysine (PLL) vs. Laminin/PLO), and culture duration. Additionally, NSPCs at different development stages may express molecules that directly influence their lineage commitment. Following exposure to Poly I:C, astroglial lineage formation was enhanced compared to neural lineage formation, as indicated by an increase in GFAP-positive cells and a decrease in ßIII-tubulin-positive cells over time. At 7 DIV, NSPC cultures treated with 10 µg/ml Poly I:C exhibited the highest percentage of GFAP-positive cells (72%). During early brain development, TLR3 is expressed not only in microglia but also in NSCs. Its expression peaks at E12.5, and decreases by E17.5, correlating with the reduction in NPC expansion (Lathia *et al*. 2008). TLR3 has been identified as a negative regulator of NSPC proliferation in the developping brain (Lathia *et al*. 2008; Yaddanapudi *et al*. 2011) and axonal growth (Cameron *et al*. 2007; Wegrzyn *et al*. 2021). Moreover, Poly I:C injection at GD 16 was shown to inhibit the transition of RGCs to IPCs, thereby reducing cortical NPC expansion (De Miranda *et al*. 2010). In contrast, a previous study demonstrated that exposing adult murine NPCs isolated from the dentate gyrus to a Poly I:C concentration of 100 µg/ml led to the upregulation of IRF3, CXCR4, and IFNaR revealing a pro-proliferative effect of TLR3 signaling on cultured NPCs (Melnik *et al*. 2012). Our findings show that the activation of TLR3 promotes NSPC differentiation into astrocytes in a time dependent manner, with the highest percentage of astrocytes observed after a longer culture period at both concentrations of Poly I:C. Further studies are needed to identify the molecules produced by NSPCs following a Poly I:C challenge.

In contrast, co-culturing NSPCs with untreated SIM-A9 cells at both 3 and 7 DIV significantly increased the number of neurons. Under these conditions, neurons outnumbered astrocytes, suggesting that SIM-A9 cells positively influence NSPC differentiation into neurons. Similarly, a previous study reported that culturing NSCs (from E16-17) with conditioned media from BV2 or N13 microglial cells, but not astroglial C6 cells, increased the proportion of neurons derived from NSCs (Aarum *et al*. 2003). Interestingly, Poly I:C-stimulated SIM-A9 cells also promoted neuronal differentiation and survival for at least 7 d, however, to a lesser extent than untreated SIM-A9 cells. This reduction aligns with the observed inhibitory effect of Poly I:C on NSPCs alone. Neverseless, the percentage of astrocytes remained higher than that of neurons.

It has been postulated that acutely activated microglia are detrimental for cell survival and neurogenesis (Cacci *et al*. 2008). Pro-inflammatory cytokines are up-regulated, NO production is enhanced and anti-inflammatory cytokines are down-regulated, creating a toxic mileu for surrounding cells. We found that some of these mediators were expressed and secreted by SIM-A9 cells upon stimulation with Poly I:C. These inflammatory mediators can induce the neurotoxic astrocytic A1 phenotype, which triggers a secondary inflammatory response. A1 astrocytes secrete CC-chemokine ligand (CCL) 2, chemokine (C-X-C motif) ligand** (**CX3CL) 1, CXCL10, and granulocyte–macrophage colony-stimulating factor (GM-CSF), which activate pro-inflammatory microglia in a feedback loop (Kwon and Koh 2020). A previous study demonstrated that culturing RGCs with A1 reactive astrocyte-conditioned media led to the death of RGCs in a concentration dependent manner. At the highest concentrations, almost all cells died. A1 reactive astrocytes can rapidly induce the death of RGCs, a subset of CNS neurons, as well as mature oligodendrocytes (Liddelow *et al*. 2017). Additionally, astrocytes themselves express TLR3 (Farina *et al*. 2005), and its activation by Poly I:C induces the mRNA expression of various mediators, including IL-1α, IL-1β, IL-6, TNF-α, GM-CSF, lymphotoxin (LT) β, and transforming growth factor- (TGF) β3, as well as the production of NO (Krasowska-Zoladek *et al*. 2007). The inflammatory response peaks within 24 h after Poly I:C binds to its receptor. However, repeated daily stimulation reduces mediator production, suggesting the presence of inhibitory feedback mechanisms that limit the pro-inflammatory response of astrocytes. The mechanisms underlying the switch from the A1 to the A2 phenotype remain unclear. Similarly, it has been shown that chronically activated microglia, such as those stimulated with LPS, express lower levels of pro-inflammatory cytokines (e.g., IL-1ß, IL-1α, IL-6, TNF-α) and NO, while producing higher levels of the anti-inflammatory cytokine IL-10 compared to acutely activated microglia (Ajmone-Cat *et al*. 2003; Cacci *et al*. 2008). Furthermore, repeated exposure to stimulating agents, like LPS, can induce immune tolerance in microglia in a dose dependent manner (Lajqi *et al*. 2019). In our study, SIM-A9 cells were chronically activated with daily media changes. Based on these findings, we speculate that chronic activation of SIM-A9 cells with Poly I:C may progressively reduce their production of pro-inflammatory molecules, increase the production of anti-inflammatory mediators and neurotrophines, and aquire a phenotype that could support neuron generation from NSPCs and be beneficial, or at least nondetrimental, to their survival. Additionally, the neuroprotective and anti-inflammatory environment in the co-culture system could trigger the switch from A1 reactive astrocytes to A2 astrocytes, promoting the neuronal survival. In parallel, repeated binding of Poly I:C to its receptor in astrocytes could make the cells less responsive to the ligand and trigger the activation of inhibitory feedback mechanisms, gradually reducing the inflammatory response.

Ultimately, Farrell *et al*. (2016) reported that co-culturing NSCs (E10) with LPS-activated SIM-A9 cells over 10 DIV resulted in enhanced astrocyte lineage commitment. Interestingly, NSPCs derived from neonatal mice (P1-3) exposed to 1 ng/ml TNF-α showed increased cell proliferation, while exposure to higher doses (10 and 100 ng/ml) induced apoptotic cell death (Bernardino *et al*. 2008). Altogether, these findings highlight that both the concentration and nature of mediators in the NSPC environment are critical factors that influence their fate.

Finally, this work requires a more detailed analysis, as it does not provide comprehensive data on the specific molecules secreted by NSPCs and SIM-A9 cells after Poly I:C stimulation. While some findings are presented here, a large-scale analysis is needed to fully characterize the secreted factors throughout the culture period. Once the inflammatory mediators are identified, further analysis should focus on determining their individual effects on the fate of NSPCs. Moreover, although neurogenesis and astrogliogenesis were examined, this study did not assess oligodendrogenesis. Future immunocytochemical analyses using markers such as Olig2 and NG2 (for progenitor and pre-oligodendrocyte stages), and MBP (for mature oligodendrocytes), could provide a more complete picture of differentiation potential of NSPCs under inflammatory conditions**.** Additionally, a key limitation of the present study lies in the use of a Transwell co-culture system. While this approach effectively isolates the effects of microglia-secreted factors, it does not allow for the direct modulation of NSPCs through physical contact with microglia. Future studies employing direct co-culture systems or three-dimensional (3D) models, such as brain organoids incorporating microglia, may offer a more comprehensive understanding of microglia/NSPC interactions under both physiological and inflammatory conditions.

In summary, these findings highlight the role of microglia in influencing the fate of NSPCs. Untreated SIM-A9 cells enhanced the differentiation of NSPCs into neurons. In contrast, Poly I:C-treated SIM-A9 cells reduced neural differentiation while still maintaining it to some extent and significantly promoted astrocyte differentiation, particularly in prolonged cultures. These changes in the neuron-to-astrocyte ratio could negatively impact brain development and contribute to neurodevelopmental disorders. The experimental model used here, co-culturing NSPCs with SIM-A9 cells, provides valuable insights into how inflammation during early development stages affects neural differentiation, potentially leading to brain diseases.

## Data Availability

Data will be made available upon request.
